# Plant-based diet in hyperkalemic chronic kidney disease patients receiving sodium zirconium cyclosilicate: a feasibility clinical trial

**DOI:** 10.1016/j.ajcnut.2024.06.025

**Published:** 2024-07-18

**Authors:** Carla Maria Avesani, Olof Heimbürger, Charlotta Rubin, Torsten Sallstrom, Gerd Fáxen-Irving, Bengt Lindholm, Peter Stenvinkel

**Affiliations:** 1 Division of Renal Medicine, Baxter Novum, Department of Clinical Science Intervention and Technology. Karolinska Institutet, Solna, Sweden; 2 Medical Unit of Clinical Nutrition, Karolinska University Hospital, Stockholm, Sweden; 3 Department of Dietetics, Nyköping Hospital, Nyköping, Sweden; 4 Division of Clinical Geriatrics, Department of Neurobiology, Care Sciences and Society, Karolinska Institutet, Solna, Sweden

**Keywords:** chronic kidney disease, dietary potassium, plant-based diet, hyperkalemia, potassium binders

## Abstract

**Background:**

Plant-based diets (PBD) may induce hyperkalemia in chronic kidney disease (CKD) patients.

**Objectives:**

We explored the safety and feasibility of PBD in hyperkalemic CKD patients receiving the potassium binder sodium zirconium cyclosilicate (SZC).

**Methods:**

In the current 6-wk trial, 26 hyperkalemic patients with CKD stage 4–5 not on dialysis received a low-protein low-potassium diet plus SZC for 3 wk and then a PBD with high potassium content delivered as a weekly food basket while continuing SZC for subsequent 3 wk. Plasma potassium was monitored weekly and SZC was titrated to achieve normokalemia. The 24-h urine excretion of potassium and sodium, 24-h food records, dietary quality, nutritional status, Bristol stool scale, Quality of life (QoL), and renal treatment satisfaction were assessed at baseline (week 0), week 3, and week 6.

**Results:**

Mean plasma potassium decreased from 5.5 to 4.4 mEq/L within 48–72 h after baseline, then rose to 4.7–5.0 mEq/L throughout the remaining study period following dose adjustments of SZC that matched the increased potassium intake of PBD from week 3 to week 6. Over the study period, 24-h urinary potassium excretion decreased from week 0 to week 3 and increased from week 3 to week 6. During the study, 58% of patients had fasting plasma potassium between 3.5 and 5.0 mEq/L and there was no episode of plasma potassium >6.5 mEq/L or <3.0 mEq/L during the study. P-carbon dioxide increased from baseline until week 6 (21 ± 2 to 23 ± 2 mEq/L; *P* = 0.002; mean ± SD), whereas remaining laboratory values remained unchanged. Fiber intake, dietary quality, the domain physical functioning from QoL, and 1 question of renal treatment satisfaction improved, whereas stool type and frequency did not change after starting PBD.

**Conclusions:**

PBD in hyperkalemia-prone CKD patients receiving SZC improved dietary quality and increased the intake of healthy foods, whereas plasma potassium concentration remained stable within normal values for most patients.

**Trial registration number:**

This trial was registered at the https://clinicaltrials.gov/study/NCT04207203 as NCT04207203.

## Introduction

Epidemiological studies show that plant-based diets (PBD) can protect against chronic kidney disease (CKD), decrease the progression of the disease, and reduce mortality risk [[1], [2], [3], [4], [5]]. PBD are defined as a dietary pattern with a predominance of plant foods (such as fruits, vegetables, legumes, and whole grains) and a lower proportion of animal proteins. Dietary patterns aligned with PBD include the Mediterranean diet, the DASH (Dietary approach to stop hypertension) diet, and the Okinawan diet, among others [4]. Of note, these dietary patterns are characterized by having increased potassium content. More recently, a higher potassium intake was shown to be associated with a lower risk of developing CKD in a population-based cohort study [6]. In light of these findings, the latest National Kidney Foundation Kidney Disease Outcomes Quality Initiative (NKF KDOQI) guideline for nutrition in CKD [7], and the Kidney Disease Improving Global Outcomes 2024 clinical practice guideline for managing CKD [8] recommended the use of PBD with healthy and diverse diets for individuals with CKD. Healthy and diverse diets comprise those with predominance of fresh foods (such as fruits, vegetables, legumes, meats, and herbs), foods with minimal degree of industrial processing for pasteurizing or drying (for example, milk and yogurt and dried herbs), ingredients used for domestic cooking (for example, vegetable oils and salt), and processed food for prolonging shelf-life but with no cosmetic additives (for example, canned corn, beans, and others). Ultraprocessed foods, that is, food that passed by high degree of industrial processing with the inclusion of cosmetics additives, sugar, hydrogenated fat, and artificial noncaloric sweeteners are to be limited (for example, potato chips, industrialized biscuits, sugary drinks, and ready-to-eat meals). The same guidelines also recommended increasing the intake of fruits and vegetables to reduce net endogenous acid production (NEAP) and possibly also the decline rate of kidney function [7,8]. However, prescribing PBD to patients with CKD and concomitant hyperkalemia is made difficult due to the high potassium content of certain healthy foods such as fruits, vegetables, legumes, whole cereals, grains, and nuts.

In addition, from a metabolic point of view, a diet restricted in healthy plant-based foods can lead to various complications such as dysbiosis and intestinal constipation [[9], [10], [11]]; worsening metabolic acidosis [12,13]; aggravation of subclinical inflammation due to the translocation of polysaccharides in the gut to the circulation [14]; increasing production of uremic toxins produced in the gut such as trimethylamine N-oxide, and indoxyl-sulfate and p-cresyl sulfate [11,15,16]; and accelerated loss of kidney function with development of other metabolic complications, such as mineral and bone disorders [13].

Considering that sodium zirconium cyclosilicate (SZC) has been shown to effectively decrease plasma potassium in patients with CKD stages 4–5 with hyperkalemia [17,18], we performed a clinical trial to test the safety of introducing a healthy PBD in patients with CKD 4–5 with hyperkalemia who received SZC to allow them to eat a less restrictive diet. Because this is the first study testing the safety of such intervention, we designed it as a feasibility study with close control of plasma potassium and other safety markers to explore the safety of this treatment approach. Concomitantly, we investigated the impact of the healthy PBD combined with SZC in controlling plasma potassium while maintaining the dosage of drugs blockading the renin-angiotensin-aldosterone system (RAAS). Quality of life (QoL) and patient satisfaction with the treatment were also assessed for evaluating the impact on patient-centered outcomes.

## Methods

### Study design and patients

This 6-wk feasibility single-arm open-label interventional trial aimed to test if a healthy potassium-rich diet with concomitant use of SZC can be safely prescribed to patients with CKD 4–5 and hyperkalemia. Based on the eligibility criteria, we included patients with a diagnosis of CKD with estimated glomerular filtration rate <29 mL/min/1.73 m^2^ not on dialysis and with hyperkalemia (5.1–6.5 mEq/L), or with normokalemia and use of sodium polystyrene sulfonate (SPS). For the latter group, SPS was discontinued for 1 wk and only then, the patient had the screening visit. Those who developed hyperkalemia were then included. Exclusion criteria included severe hyperkalemia (>6.5 mEq/L), probable start of dialysis in 2 mo according to the nephrologist´s clinical judgment, presence of inflammatory bowel syndrome, history of hypokalemia (<3.0 mEq/L), and lack of fluency in Swedish. The patients were recruited at the specialized CKD outpatient clinics at Karolinska University Hospital. All patients who fulfilled the eligibility criteria were accepted to be enrolled in the study. This study was registered at www.clinicaltrials.gov (NCT04207203) on December 19, 2019. The study was approved by the Swedish Ethical Review Authority (registration number 2019-05432, January 18, 2020) and by the Swedish Medical Product Agency (registration number 5.1-2020-39451, June 16, 2020). The study was conducted in accordance with the Helsinki Declaration.

### Study procedures and intervention

**FIGURE 1:**
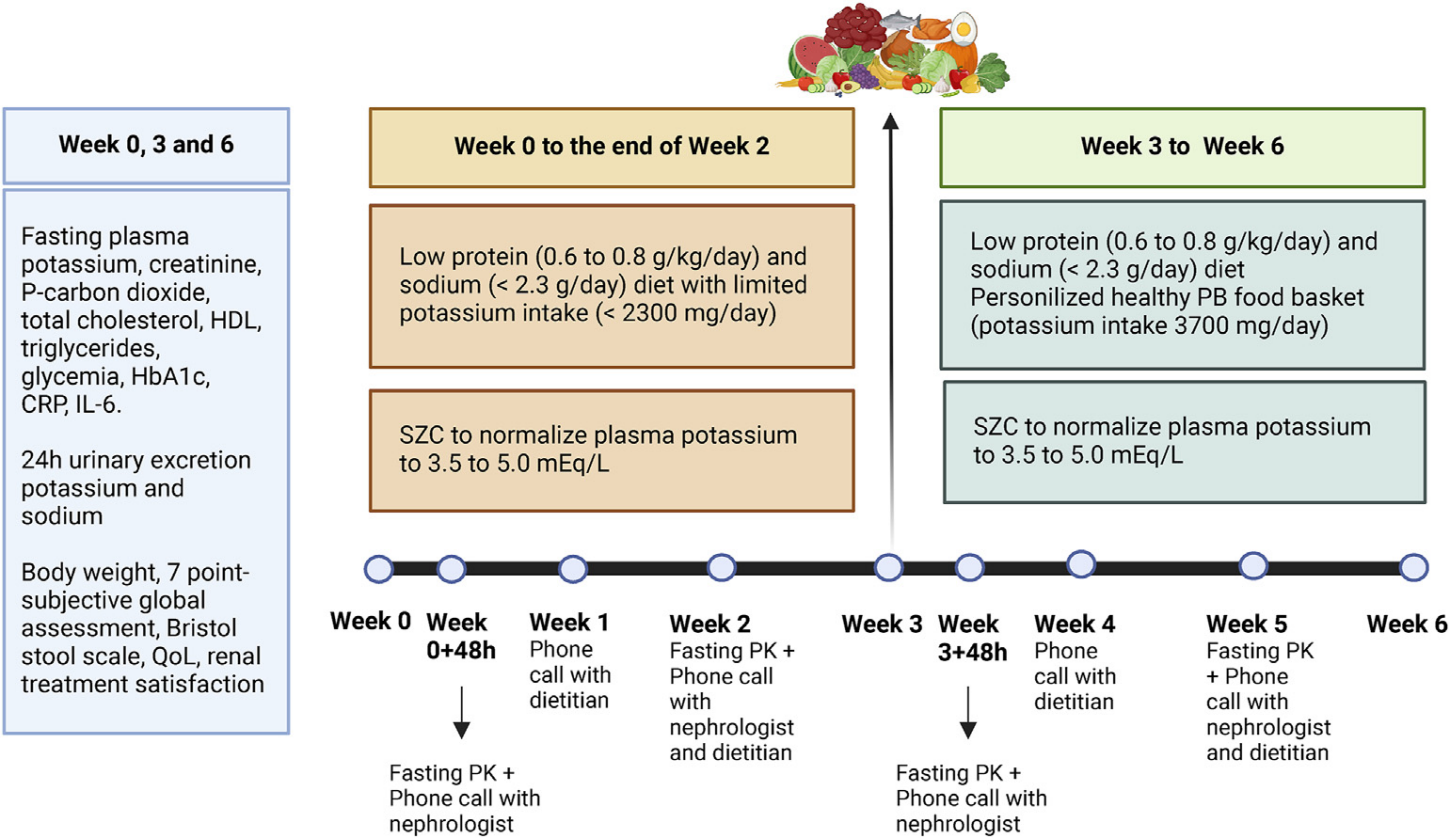
Study design and procedures (*n* = 26). CRP, C-reactive protein; HbA1c, glycated hemoglobin; PB, plant-based; PK, plasma potassium; QoL, Quality of Life; SZC, sodium zirconium cyclosilicate.

The study lasted 6 wk, encompassing a 3-wk phase to normalize plasma potassium followed by a 3-wk healthy PBD phase. Figure 1 illustrates the study design and procedures. The patients visited the research clinical unit at week 0, week 3, and week 6 for laboratory examinations, nutritional status, evaluation of stool type, frequency (Bristol stool scale [19]), and patient-centered parameters (QoL and satisfaction with the treatment). For that, the HAND-36 QoL questionnaire and the Renal Treatment Satisfaction Questionnaire, both validated for patients with CKD in Sweden, were used in this study [20,21]. Additional fasting plasma potassium measurements were performed 48 h after the inclusion in the study (week 0), on week 2, 48 h after the start of the healthy PBD food basket (week 3), and on week 5, in the laboratory connected to the university hospital closer to the patient’s house complemented with a telephone call by the research nurse, nephrologist, and dietitian for monitoring potassium medication and the adherence to the diet. During all 6 wk, patients used SZC to control plasma potassium. SZC was dosed as indicated on the label and was used as described in Supplemental Tables 1, **2**. In accordance with the medication label, in the first 48–72 h of the study, the patients received 30 g/d of SZC and once plasma potassium was normalized the dose was adjusted to 5–10 g/d depending on the fasting plasma potassium concentration, which was titrated by the nephrologist to keep the fasting plasma potassium between 3.5 and 5.0 mEq/L (Supplemental Table 2). In addition, on week 0, all patients received an individualized dietary plan advised by a renal dietitian that consisted of a low-protein diet (0.6–0.8 g/kg/d) with low potassium (<2300 mg/d) and sodium (<2.3 g/d) content, as advised in the Nutrition NKF KDOQI guideline [7]. On the first day of week 3, the dietitian advised the patients to increase the intake of potassium to 3700 mg/d. For increasing adherence, a food basket containing 3 servings of fruits, 2 of vegetables, 2 of legumes, 1 of nuts, 4 of whole grains, and 2 of poultry, fish, or egg per day (Supplemental Table 3) was delivered once per week to the patient’s household with food for the patient and every adult living in the same household. The food basket was individualized according to the patient’s food preference and possible food allergies, but the number of servings of each food group was the same for all patients. Particularly for fish, poultry, or eggs, the number of servings in the food basket was the same for all patients, but instruction on the amount that the patient would eat was individualized according to the patient’s body weight. Patients were allowed to eat food outside the food basket, but were instructed to limit the intake of ultraprocessed food such as ready-to-eat meals, processed meat, chips, industrialized juices, flavored teas, and coffee drinks. No restriction was given regarding the intake of nonprocessed tea, coffee, and juices from fresh fruits. Forty-eight hours after the start of the healthy PBD, the patient had a new fasting plasma potassium measurement, and the nephrologist adjusted the SZC if needed, according to Supplemental Table 4. Safety procedures were carried out at the inclusion and during the 6-wk study period. Detailed descriptions of the intervention, methods, and safety procedures can be found in the Supplementary material.

### Statistical analysis

Continuous variables are shown as mean and SD or as median and IQR. Categorical variables are shown as absolute numbers and percentages. We used repeated measures analysis of variance (ANOVA) tests for normally distributed variables and related sample Friedman’s tests for nonnormally distributed variables to compare changes between the time points during the study. Post hoc analysis with Bonferroni correction for repeated measures ANOVA or Friedman 2-way ANOVAs by ranks for related samples for multiple comparisons were performed when the ANOVA or Friedman’s test *P* value was <0.05. The measurements at the different time points were registered for all patients and tested in most cases with no missing data. The only variables with missing data were QoL (in 1 patient) and Bristol scale (in 9 patients) and in these cases, the analysis that had rows with missing data were excluded. All tests were performed using the statistical software IBM SPSS Inc. (Statistical Package for the Social Sciences) (version 28.0.0.0). A *P* value <0.05 was considered statistically significant.

## Results

The inclusion, intervention, and follow-up occurred between November 2020 and May 2023, with the date for the first patient visit on the first week of November 2020, the last patient first visit on the second week of April 2023, and the last patient’s last visit on the last week of May 2023. This is a convenience sample. We planned to include 36 patients monitored at the CKD outpatient clinic at the Karolinska University Hospital who fulfilled the eligibility criteria. Due to the COVID-19 pandemic that imposed restrictions on the inclusion of patients and due to difficulties in finding patients within the fasting plasma potassium ≥5.1 mmol/L and <6.0 mmol/L on the day of inclusion, 26 patients were enrolled. As shown in Supplemental Figure 1, from the total number of screenings, 22 patients were included with no rescreening and 4 patients were included after 1 rescreening due to plasma potassium <5.1 mEq/L on the first screening visit. All included patients completed the 6-wk study protocol.

### Main characteristics at baseline

As described in Table 1, the mean age at baseline was in the sixth decade of life, and about half of the patients were males; 17 patients had CKD stage 4 and 9 patients had CKD stage 5. Most of them (*n* = 20; 77 %) were previously using SPS before their inclusion in the study. The most frequent diagnoses of causes for CKD were IgA nephropathy, diabetic nephropathy, and glomerulonephritis. The nutritional status assessed by 7 point-subjective global assessment showed that at baseline the majority was well-nourished and the mean BMI (kg/m^2^)was indicative of overweight.

**TABLE 1:** Main characteristics at baseline (week 0) of the included patients with chronic kidney disease (*n* = 26).

	Included sample^1^
Age (y)	61 ± 13
Male (*n*; %)	17; 65.4%
CKD stage	
Stage 4	17 (65.4%)
Stage 5	9 (34.6%)
Hemoglobin (g/L)	122 ± 16
Serum albumin (g/dL)	3.50 ± 0.3
Spot urinary albumin creatinine ratio (mg/g)	592 (115; 1043)
Previous use of SPS (*n*; %)	20; 76.9%
CKD etiology	
IgA nephropathy	6 (23%)
Diabetic nephropathy	5 (19%)
Glomerulonephritis	3 (12%)
Others	12 (46%)
Nutritional status^2^	
Well-nourished	19 (73%)
Risk of malnutrition	6 (23%)
Mild malnutrition	1 (4%)
Body mass index (kg/m^2^)	27.5 ± 4.0

Abbreviations: CKD, chronic kidney disease; IgA, immunoglobulin A; SPS, sodium polystyrene sulfonate.

1 Data are presented as mean ± SD or median and IQR or as number and percentage, as appropriate.

2 Assessed by 7 point-subjective global assessment.

### Potassium control

**FIGURE 2:**
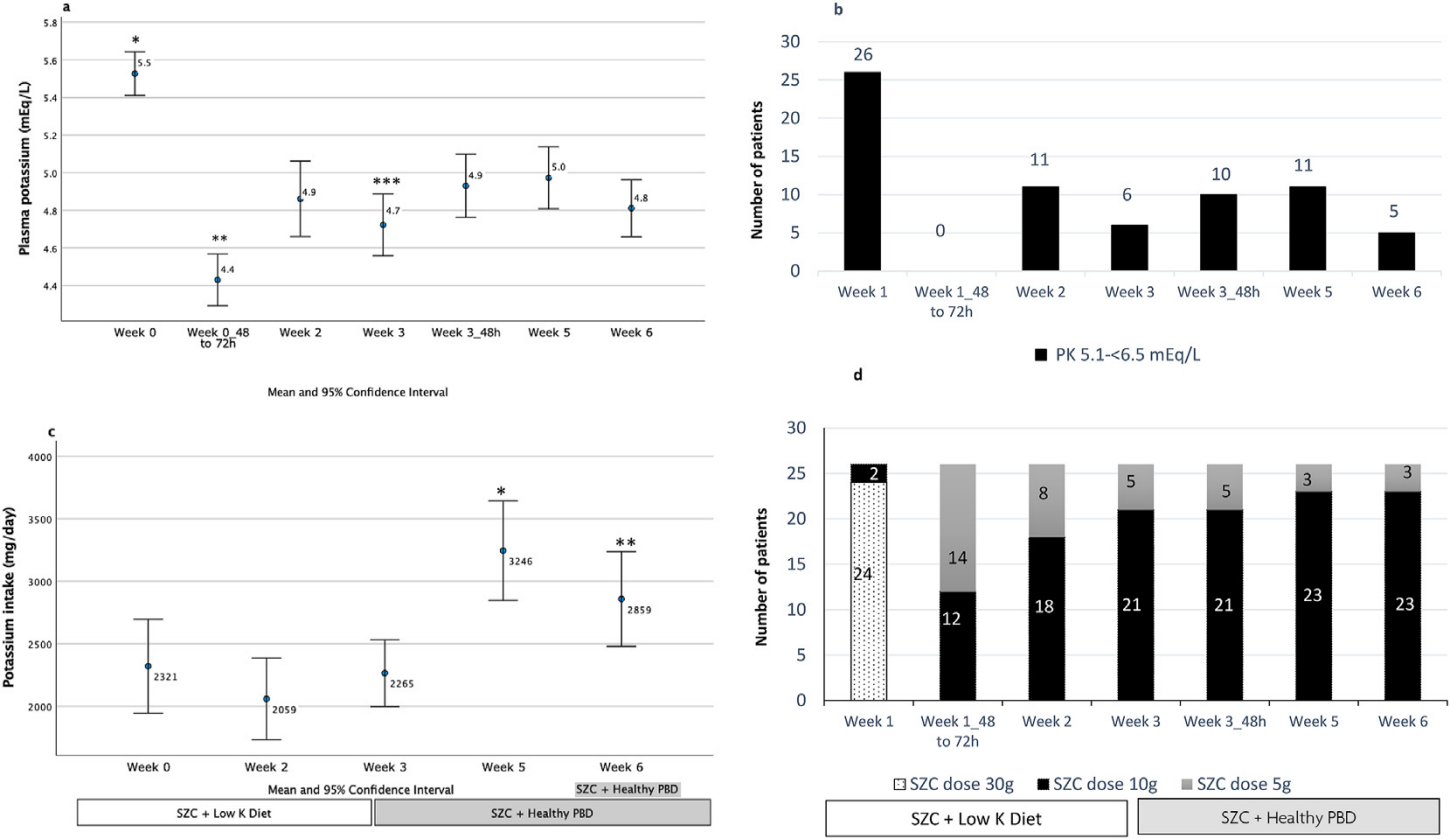
Potassium control during the study (*n* = 26). (A) Fasting plasma potassium (*n* = 26); repeated measure ANOVA test (*P* < 0.01). Post hoc analysis with Bonferroni correction for multiple comparisons: ∗Indicates *P* < 0.05 for the comparison between week 0 to all other time points. ∗∗Indicates *P* < 0.05 between week 0_48–72 h and all time points. ∗∗∗Indicates *P* < 0.05 between week 3 and week 3_48 h. (B) Number of patients with hyperkalemia (plasma potassium: 5.1 to <6.5 mEq/L) (*n* = 26). (C) Potassium intake assessed by 24-h food record (*n* = 26). Repeated measure ANOVA test (*P* < 0.01). Post hoc analysis with Bonferroni correction for multiple comparisons: ∗Indicate *P* < 0.05 for the comparison between week 5 to all other time points. ∗∗Indicate *P* < 0.05 between week 6 and all time points. (D) SZC prescribed daily dose (*n* = 26). ANOVA, analysis of variance; SZC, sodium zirconium cyclosilicate.

Figure 2 describes results related to potassium control during the study. Mean plasma potassium decreased after 48–72 h (5.5–4.4 mEq/L) of using the higher dose of SZC and was kept within normokalemia (3.5–5.0 mEq/L) (Figure 2A), but with some patients in the range of hyperkalemia (5.1–6.5 mEq/L) (Figure 2B), with maximum 5.9 mEq/L 48 h after start of the PBD (Supplemental Table 5). The potassium intake as assessed by the food diaries increased on week 5 and week 6 as compared with other time visits (Figure 2C). When assessed by the 24-h urinary potassium excretion, the potassium intake decreased from week 0 to week 3 and increased from week 3 to week 6 (Table 2). For both methods, potassium intake increased on week 6 as compared with week 3. The most frequently prescribed dose of SZC was 10 g/d, mainly after the start of the PBD (Figure 2D). From 48 to 72 h onward, 58% of the patients had fasting plasma potassium between 3.5 and 5.0 mEq/L during the remaining 6-wk study period.

**TABLE 2:** Laboratory examinations during the study of the included patients with chronic kidney disease (*n* = 26).

	Week 0 baseline^1^	Week 3 before PBD^1^	Week 6 end PBD^1^	*P*^2^
Creatinine (mg/dL)	3.7 ± 1.0	3.5 ± 1.1	3.6 ± 1.1	0.89
eGFR (mL/min/1.73 m^2^)	17 (14; 20)	17.5 (14.7; 21.3)	17 (13.8; 21)	0.30
Urea (mg/dL)	114 ± 36	102 ± 30	108 ± 36	0.08
P-carbon dioxide (mmol/L)	21 ± 2	22 ± 2	23 ± 2^3^^,^^4^	0.002
Phosphorous (mg/dL)	4.2 ± 1.0	4.0 ± 1.0	4.0 ± 0.9	0.48
C-reactive protein (mg/L)	2.0 (1.0; 3.0)	1.0 (0.9; 3.0)	2.0 (1.5; 3.5)	0.82
Interleukin-6 (pg/mL)	2.9 (2.0; 8.0)	3.6 (2.0; 6.4)	2.9 (2.0; 5.9)	0.49
Total cholesterol (mg/dL)	185 ± 50	189 ± 50	189 ± 46	0.76
LDL cholesterol (mg/dL)	104 ± 38	104 ± 42	104 ± 42	0.24
HDL cholesterol (mg/dL)	50.3 ± 15.6	58 ± 23	50.3 ± 15.5	0.08
Triglycerides (mg/dL)	159 ± 88	159 ± 79	168 ± 97	0.32
Fasting glucose (mg/dL)	106 ± 27	103 ± 23	106 ± 25	0.78
HbA1c (%)	5.9 ± 3.2	5.8 ± 3.2	5.8 ± 3.2	0.30
24-h urinary potassium excretion (mmol/d)	49 (42; 64)	41 (35; 50) 3^,^^5^	48 (31; 66) 3^,^^6^	0.02
24-h urinary sodium excretion (mmol/d)	184 (127; 223)	180 (144; 250)	205 (138; 242)	0.43

Abbreviations: ANOVA, analysis of variance; eGFR, estimated glomerular filtration rate; HbA1c, glycated hemoglobin; PBD, plant-based diet.

1 Data are presented as mean ± SD or as median (IQR).

2 Repeated measures ANOVA or related sample Friedman’s test, as appropriate.

3 Post hoc analysis with Bonferroni correction for repeated measures ANOVA or 2-way ANOVAs by ranks for related samples by Friedman’s test for multiple comparisons.

4 Indicates *P* < 0.05 for the comparison between weeks 6–0 and 3.

5 Indicates *P* < 0.05 for the comparison between weeks 3 and 0.

6 Indicates *P* < 0.05 for the comparison between weeks 6 and 3.

### Blood markers and dietary intake

Laboratory parameters are shown in Table 2. Except for P-carbon dioxide that significantly increased on week 6 as compared with week 0 and week 3, the remaining laboratory values remained unchanged. Regarding the dietary intake (Table 3), after the PBD, there was a significant increase in fiber intake, in the mean adequacy ratio (MAR) of the intake of 11 nutrients as compared with the recommended daily intake (RDI) and in the intake of fruits, vegetables, and nuts. The intake of red meat decreased, whereas that of poultry and fish increased. In addition, the Bristol stool scale showed no significant changes in frequency or type of stool during the study (data not shown).

**TABLE 3:** Data related to food intake during the study of the included patients with chronic kidney disease (*n* = 26).

	Week 0 baseline^1^	Week 3 before PBD^1^	Week 6 end PBD^1^	*P*^2^
Energy intake (kcal/kg^3^/d)	21.1 ± 6.6	21.5 ± 6.6	25.2 ± 8.9	0.44
Protein intake (g/kg^3^/d)	0.73 ± 0.24	0.71 ± 0.20	0.80 ± 0.23	0.30
Phosphorus intake (mg/d)	950 ± 383	890 ± 251	1064 ± 341	0.062
Fiber intake (g/d)	19.2 ± 7.0	19.3 ± 7.0	24.7 ± 9.0^4^^,^^5^	0.002
S-NRF11.3	34 (28;42)	35 (25; 42)	40 (27; 48)	0.19
MAR-11, %	77 (70; 83)	72 (67; 84)	86 (73; 90)^4^^,^^5^	0.021
Fruits (serving/d)	1.5 (1.0; 2.5)	1.5 (1.0; 2.1)	3.4 (2.5; 4.2)^4^^,^^5^	<0.01
Vegetables (serving/d)	1.0 (0.7; 2.0)	1.5 (0.9; 2.0)	2.0 (1.0; 2.6)^4^^,^^5^	0.04
Nuts (serving/wk)	0.1 (0; 4.8)	1.0 (0; 5.5)	11.0 (6.5; 22.0)^4^^,^^5^	<0.01
Whole cereals (serving/wk)	13.0 (3.4; 20.0)	8.5 (1.9; 14.5)	14.0 (8.0; 20.2)	0.28
Red meat (serving/wk)	1.4 (0.8; 2.1)	2.0 (1.0; 3.0)	1.0 (0.3; 2.0)^4^^,^^6^	0.03
Poultry (serving/wk)	1.0 (0.5; 2.0)	1.5 (1.0; 3.0)	2 (1.0; 3.0)^4^^,^^7^	0.02
Fish (serving/wk)	1.3 (1.0; 2.6)	1.5 (1.0; 2.0)	2.3 (2.0; 3.4)^4^^,^^5^	<0.001
Egg (serving/wk)	2.0 (0.5; 3.5)	2.5 (1.0; 4.2)	2.3 (1.0; 6.6)	0.17

Abbreviations: ANOVA, analysis of variance; MAR, mean adequacy ratio; PBD, plant-based diet; S-NRF11.3, Swedish version of Nutrient rich food index.

1 Data described as mean ± SD or as median (IQR).

2 Repeated measures ANOVA or related sample Friedman’s test, as appropriate.

3 Actual body weight.

4 Post hoc analysis with Bonferroni correction for repeated measures ANOVA or 2-way ANOVAs by ranks for related samples by Friedman’s test for multiple comparisons.

5 Indicates *P* < 0.05 for the comparison between weeks 6–0 and 3.

6 Indicates *P* < 0.05 for the comparison between weeks 6 and 3.

7 Indicates *P* < 0.05 for the comparison between weeks 6 and 0.

### Patient-centered outcome and gastrointestinal symptoms

The QoL showed improvement in the domain of physical activity and the question related to the satisfaction to continue with the treatment also ameliorated at the end of the study (week 6) as compared with baseline (week 0) (Supplemental Tables 6, 7). Regarding gastrointestinal symptoms, 10 mild and transient events were registered including constipation, intermittent nausea, abdominal distention, stomach ache, mild diarrhea, and symptoms of hemorrhoids. Constipation was the most frequent symptom and was often present before the initiation of PBD. In the physician’s assessment, the relation of the gastrointestinal symptoms with the use of SZC was rated as “probable” or “possible” in 6 events. A gout complaint was reported by 1 patient and was not rated as related to the medication according to the physician´s assessment.

### Dosage of RAAS blockers

The dosage of RAAS blockers remained unchanged during the study, with 13 patients using an angiotensin-converting enzyme inhibitor and 12 patients using an angiotensin receptor blocker.

### Safety measures

Plasma potassium was kept within normal values 48–72 h after the start of SZC with no episodes of hyperkalemia reported. There was no episode of plasma potassium >6.5 mEq/L or <3.0 mEq/L nor any case of QTc > 550 ms on the echocardiogram during the study.

## Discussion

Whereas the use of the new potassium binders in the CKD population to safely and effectively managing hyperkalemia is thought to have the potential to allow a more liberal and healthy diet in patients with CKD at risk of or with hyperkalemia [[22], [23], [24], [25], [26]], this has not been demonstrated previously. We present for the first time the results of a study exploring this strategy and showing that this approach seems feasible and safe. To ensure safety, we designed a 6-wk-long feasibility study with close and weekly follow-up of changes in plasma potassium for 3 wk before and 3 wk after introducing a healthy PBD in patients with CKD and hyperkalemia who were receiving treatment with SZC. The order by which the type of diet was chosen could not be randomized for safety reasons because the patients had to have hyperkalemia at inclusion. Therefore, the intervention started with the prescription of SZC and low-potassium diet for the first 3 wk. In the next 3 wk, a diet with an increased amount of potassium was prescribed while maintaining SZC. Our study shows that 58% of the patients had fasting plasma potassium within the normal range between 3.5 and 5.0 mEq/L during the entire 6-wk study period and no episodes of severe hyperkalemia.

The main purpose of this study was to investigate the effects of an increased dietary potassium intake by introducing a PBD with higher potassium content that is thought to represent a healthier diet than that of a restricted potassium intake. All participants received weekly food baskets containing their preferred fruits, vegetables, legumes, nuts, whole cereals, and white meat or eggs, not restricting their choice due to potassium content. Patients adhered to the prescribed diet as confirmed by the increase in potassium intake assessed by the 24-h food records and by the 24-h urine potassium excretion. During the 3-wk period with the PBD (weeks 3–6), the mean plasma potassium was normal, but 5–11 patients (19%–38%) had 1 or more episodes of mild to moderate hyperkalemia with plasma potassium between 5.1 and 5.9 mEq/L. Our study demonstrates that the explored approach is feasible and safe, thus paving the way for future randomized controlled trials testing the long-term safety of healthier diets and also exploring the effect of those diets in ameliorating metabolic outcomes.

The finding that plasma potassium did not significantly change after the increase in potassium intake goes in the same direction as in other studies that explored if potassium intake needs to be restricted to patients with CKD. Observational studies showed that potassium intake (assessed by food records or by 24-h urine potassium excretion) was not associated (or was weakly associated) with plasma potassium in patients with CKD including those on hemodialysis [[27], [28], [29], [30], [31]]. Complementing these findings, 2 randomized controlled studies prescribing an increase in the intake of fruits and vegetables to patients with CKD 3–4 for longer periods (3 y and 26 wk, respectively), the plasma potassium did not increase significantly, and no difference was observed between the group ingesting more servings of fruits and vegetables than the control group [13,32]. However, the patients had normokalemia at baseline and the prescribed diet contained 3000 mg potassium/d. In a randomized feeding trial with 2-wk period cross-over design that tested a high compared with low-potassium diet in 29 patients with CKD stage 3 and normokalemia, it was observed that during the period of the high potassium diet, the plasma potassium increased significantly and the odds of hyperkalemia (plasma potassium > 5.5 mEq/L) was 2.5 higher (95% CI: 1.04, 6.00; *P* = 0.04) than during the period with low-potassium diet [33]. In this study, even though all patients had hyperkalemia at baseline, we were able to obtain similar findings by treating them with SZC throughout the period with a more liberal diet with increased content of potassium (weeks 3–6). Of note, the use of RAAS inhibitors, which impose a risk of worsening hyperkalemia, was maintained during the study period. This adds clinical relevance to the treatment because RAAS inhibitors are mandatory for controlling blood pressure, proteinuria, and progression of the underlying disease in patients with CKD.

Concomitantly, we observed that the overall dietary quality markedly improved as depicted by a rise in MAR% (an index of adherence to the RDI), and by the significant increase in the intake of fibers, and servings of healthy foods such as fresh fruits and vegetables, and nuts without providing food additives containing potassium and phosphorous. Phosphorous intake had a borderline increase during the study that likely reflected a less restrictive diet with higher reported servings of nuts that have high phosphorous content, but with bioavailability much lower than that of inorganic phosphorous present in food additives. Protein intake did not change, there was an exchange from eating red meat to poultry and fish. Such a shift was reported to reduce the development of noncommunicable diseases and increase the 10-y life expectancy in the general population from the United Kingdom aged 40–70 y [34]. From the clinical point of view, the possibility for patients with CKD to eat a diet allowing fresh healthy foods without food additives could have several metabolic benefits. For example, the P-carbon dioxide significantly increased after the introduction of the healthy PBD. It is possible that the effect of the SZC medication could have contributed to this result because it has been shown that bicarbonate increased in a previous study that involved intervention with SZC [35]. However, if one considers that during the 6-wk period, the use of SZC did not change, the changes in the diet introduced at week 3 likely diminished NEAP contributing to the increase in P-carbon dioxide. This finding is aligned with previous studies where the increase in the intake of fruits and vegetables, but for longer periods, ameliorated bicarbonate or P-carbon dioxide in patients with CKD stages 3–4 [13,32,36].

A 3-wk period of a PBD is too short to achieve other metabolic improvements, but there may be other potential benefits, such as improvements in proteinuria, blood pressure, phosphorous excretion, and reduction in abdominal fat, as those reported previously with PBD in CKD [13]. In addition, positive effects of an amelioration in gut microbiota composition mediated by the observed higher fiber intake can also be expected [10]. Complementing these findings is the improvement in the physical activity domain of QoL and of some aspects of satisfaction with the treatment from the beginning (week 0) to the end of the study (week 6). We do not know the reason for these positive findings, but hypothesize that it can be due to a combination of factors, such as appreciation of being more closely monitored by a multidisciplinary team, receiving a more liberal diet with food reflecting their preferences, and being provided with a free food basket from the 3rd to 6th wk of the study.

This study carries strengths and limitations. The limitations include the study design which aimed to explore the feasibility of a combined strategy of medication and diet. Because there is no control group, the study does not allow extrapolating conclusions about the effect on plasma potassium of a potassium-low compared with potassium-high diet. Second, this study evaluated only fasting plasma potassium, whereas the postprandial plasma potassium control was not studied. Thus, the transitory event of postprandial hyperkalemia cannot be ruled out and remains to be investigated. Third, because the food intake prior to the enrollment was not known, we could not evaluate the acute effect of the SZC alone without the dietary intervention. Fourth, 4 patients were included after 1 rescreening due to plasma potassium <5.1 mEq/L. We cannot rule out that the plasma potassium of these patients could fall more easily during the study and be less subjected to hyperkalemia due to the use of medication or higher potassium intake. Fifth, the sample size was limited to 26 patients which may be inadequate to explore statistical differences between the study visits. Finally, the period of follow-up was limited to 6 wk, which is a too short period for exploring metabolic changes related to dietary changes. As strengths, the weekly follow-up was carried out by nephrologists, nurses, and dietitians allowing an individualized and careful control of plasma potassium ensuring patient’s safety with the treatment and adherence to diet. Second, investigating the dietary intervention using weekly food baskets for the patient and to each adult living in the same household is a strength for supporting adherence to a healthier diet for the whole family. The delivery of the weekly free food baskets according to the patients’ preferences and habits received a positive response from the participants that speaks in favor of acceptable adherence to the diet. The positive experiences suggest that this is a feasible strategy for studies of the role of dietary changes in individuals in free-living conditions as opposed to studies that have patients staying in a clinical research center for usually much shorter periods and in conditions that do not reflect their normal lives. Finally, the interest in using food as a central part of the treatment of many different diseases and conditions is increasing widely, and our study is aligned with this strategy of “Food as medicine” [13,32,[37], [38], [39], [40], [41]].

In summary, our study exploring the feasibility and safety of a PBD not restricted in potassium together with the concomitant use of SZC in patients with hyperkalemia and CKD 4–5 showed that this combined strategy allowed the majority of patients to remain normokalemic, and no patient experienced severe hyperkalemia. Other positive results included amelioration of the dietary quality with an increase in the intake of healthy foods, and amelioration of acid-base control, QoL, and renal treatment satisfaction. In a broader context, the results of the current trial suggest that CKD patients with hyperkalemia do not need restrictions on dietary potassium intake when using SZC to control plasma potassium. Prescribing a PBD to patients with CKD with possible beneficial effects on metabolism may represent an attractive paradigm shift, although to establish its role in clinical practice, a larger randomized controlled trial with a longer follow-up period is needed.

## Acknowledgments

We thank the research nurses from the Nephrology Medical Research Unit of Karolinska University Hospital, especially Sofie Garpemo, Anneli Lindberg, Awa Gaye Danielsson, and Pernilla Bolander for the careful work. Baxter Novum is the result of a grant from Baxter Healthcare to Karolinska Institutet.

## Author contributions

The authors’ responsibilities were as follows – CMA, OH, GF-I, BL, PS: designed research; CMA, OH, CR, TS, PS: conducted research; CMA: analyzed data; CMA, BL, PS: wrote article; GF-I, OH: critically revised the article; CMA, BL, PS: had primary responsibility for final content; and all authors: read and approved the final manuscript.

### Conflict of interest

CMA received speaker honorarium from Astra Zeneca, Fresenius Medical Care, Abbott, and Baxter Healthcare, and received honorarium as an advisory board member from Fresenius Kabi. OH received speaker honorarium from Astra Zeneca, Baxter Healthcare, EwiMed, and honorarium for advisory board from Opterium. BL has been supported by a grant from Baxter Healthcare to Karolinska Institutet. PS received speaker honorarium from Astra Zeneca, Baxter Healthcare, Fresenius Medical Care, Novo Nordisk, Astellas, Pfizer, and Bayer, and also received honorarium as an advisory board member from Fresenius Medical Care, Astra Zeneca, Vifor, Baxter Healthcare, Invizius, CSL, and Boehringer Mannheim. TS, CR, and GF-I declare no conflicts of interest.

## Funding

This study was supported by Astra Zeneca. The sponsor was involved in the study design, but not in the collection, analysis, and interpretation of data as well as data checking of information provided in the manuscript. In addition, the ultimate responsibility for opinions, conclusions, and data interpretation is from the authors.

## Data availability

Data described in the manuscript, code book, and analytic code will be made available upon request.
