# Supplementary material for: Plant-based diet in hyperkalemic chronic kidney disease patients receiving sodium zirconium cyclosilicate: a feasibility clinical trial
Source: Am J Clin Nutr. 2024 Jul 18;120(3):719–26. doi: 10.1016/j.ajcnut.2024.06.025 (PMC11393394; doi:10.1016/j.ajcnut.2024.06.025)
Supplement: multimedia component [file mmc1.docx]

**Supplementary material**

**Detailed methods description**

*Study procedures and intervention*

The study lasted six weeks, encompassing a 3-week phase to normalize plasma potassium followed by a 3-week healthy PBD phase. At the screening visit that occurred in the morning, written informed consent was obtained and after that, blood drawn in fasting condition was acquired for patients not using sodium polystyrene sulfonate (SPS). If all eligibility criteria were fulfilled, the patient was included in the clinical trial. If the patient was using SPS, the nephrologist instructed the patient to stop the SPS and return to the clinical research center after one week. Only then, the patient had the screening visit with the same procedure as described for the other patients. In case of screening failure due to low potassium levels, the patient could be rescreened for another two times with interval between the visits according to nephrologist discretion and as agreed with the patient.

After the patient was enrolled in the clinical trial, the patient received a light breakfast and the assessment continued with the evaluation of the nutritional status by seven point-subjective global assessment (7p-SGA), food intake assessed by 24-hour food recall and short food frequency semi-quantitative questionnaire. All patients met with an experienced renal dietitian who advised them to follow a diet with energy content of 25-35 kcal/kg/day, protein content between 0.6-0.8 g/kg/day, sodium (<2.3 g/day) and potassium (<2300 mg per day). Patients were counselled to refrain from red meat and processed meat such as sausages and ham to keep the diet within a healthy standard pattern. The dietitian provided a detailed oral and a written personalized dietary plan and a book containing recipes developed for patients with kidney failure who were following a low protein diet. The patients were instructed to start evaluating their stool in the next day according to a 7-day Bristol scale (1) and bring it back on the visit at Week 3. Once all initial procedures were concluded, the potassium binder SZC was prescribed by the nephrologist following the dosage according to the medication label. In the first 48 hours, a higher dose of SZC was prescribed (10 g, three times day) and it was then reduced to 5 to 10 g/day depending on the plasma potassium that was assessed after 48 hours following the start of SZC and if needed again in the next 24 hours (**Supplementary Table 1**). After that it was assessed again on Week 2 followed by adjustment in the SZC dosage if needed (**Supplementary Table 2**). The dietitian reached out to the patient by phone call on Week 1 and 2 for ascertaining and reinforcing adherence and checking doubts regarding the diet. In the first day of the 3^rd^ week of the protocol, the patient returned to the research center and repeated the same tests and questionnaires as were done at baseline (Week 0). In addition, the same renal dietitian advised a healthy PBD that consisted of the same prescription of energy, protein, and sodium, but with higher amount of potassium (3700 to 4000 mg of potassium/day). An individualized food basket containing known amounts of fruits, vegetables, whole grains, nuts and white meat, fish and egg according to their food preference was delivered (free of charge to the patient) once per week to the patients’ home. Each food basket contained three servings of fruits, two servings of vegetables, two servings of legumes, one serving of nuts, four of whole grains and two servings of white meat, fish or egg for the patient and for all adults living in the same household (**Supplementary Table 3**). For planning the food basket, in the first study visit, all patients completed a form with their preferences and / or allergies to the food groups that comprised the food basket. With this information, the food basket individualized according to the patient´s preference. The cost for the PBD food basket per week per capita per household was 44 ±14 €. Patients could eat foods outside the food basket.

Forty-eight hours after the start of the healthy PBD, the patient had a new fasting plasma potassium measurement, and the nephrologist adjusted the SZC if needed according to **Supplementary Table 4**. A 7-day Bristol scale was given to the patient for returning it at week-6. After that, the patient had measurements of fasting plasma potassium on week 4 and 5, and the SZC was adjusted by the nephrologist if needed (**Supplementary Table 4**). The dietitian made phone calls to the patient on Week 4 and Week 5. On the first day of Week 6, the patient returned to the research center for repeating the laboratory measurements and questionnaires from baseline and Week 3. The 7-day Bristol scale was given to be returned by regular mail. On this day the patient had the end-of-study visit. Those who wished could continue using SZC and the healthy PBD but without receiving a food basket at home. The patients continued their regular medical follow-up at the CKD outpatient clinic.

The laboratorial tests that were assessed on week 0, week 3 and week 6 included potassium, urea, creatinine, phosphorus, P-carbon dioxide, interleukin-6 (IL-6), C-reactive protein (CRP), glucose, glycated hemoglobin (HbA1c), total cholesterol, triglycerides, and HDL-cholesterol. In 24-hour collections of urine, potassium and sodium excretion were measured. The estimated glomerular filtration rate (eGFR) was calculated according to the Lund-Malmö equation (2). Hemoglobin, serum albumin and urinary spot albumin creatinine ratio were acquired from the routine exams closest to the date of inclusion in the protocol.

Two dietary quality scores were calculated based on the 24-hour food recall. The first one by using the Swedish version of Nutrient rich food index (S-NRF11.3) as a measure of the density of 11 beneficial nutrients (fibre, folate, iron, calcium, potassium, protein and vitamins A, C, D and E) and of 3 non-beneficial nutrients (saturated fat, sodium and added sugars) and the second by using the mean adequacy ratio (MAR), describing the average intake of the same 11 beneficial nutrients, as a proportion of the recommended daily intake (RDI). The MAR is reported on a scale of 1 to 100% , where higher scaled indicates that requirements for the 11 nutrients were met (3).

*Safety procedures*

All females in fertile age had pregnancy tests performed by blood beta subunit of human chorionic gonadotropin test before their inclusion. If a patient would become pregnant during the study, the investigational product would be discontinued immediately.

If normokalemia (plasma potassium between 3.5 to 5.0 mEq/L) was not achieved after 48 hours after the start of the treatment with SZC, the patient continued with 30 g of SZC and a new measurement in the subsequent 24 hours was performed. If normokalaemia was achieved, the SZC dose decreased as described in **Supplementary Table 2**. But, if normokalaemia was not achieved after the 72 hours, the patient would discontinue SZC and start rescue treatment with prescription of other potassium binders treatment (i.e., SPS) according to FASS (Farmaceutiska specialiteter i Sverige/ Pharmaceutical specialties in Sweden which is a web-based information on medicinal products in Sweden). The patient would anyhow continue in the study with the measurements of plasma potassium as described in the protocol. If hypokalemia (plasma potassium <3.0 mEq/L) would occur, the occurrence would be registered, the treatment with SZC discontinued and the patient would continue in the study. Rescue therapy would start, plasma potassium would be checked, and potassium supplemented as needed.

Electrocardiogram (ECG) was performed at baseline (Week 1), Week 3 and Week 6 according to standards procedures. If an absolute correct QT interval (QTc) >550ms, or an increase in QTc interval > 60ms from baseline to more than 500ms is reached the subject should immediately receive appropriate medical intervention and be discontinued from the study drug treatment.

**References**

1. Lewis SJ, Heaton KW. Stool form scale as a useful guide to intestinal transit time. Scand J Gastroenterol. 1997;32(9):920-4. doi: 10.3109/00365529709011203.

2. Nyman U, Grubb A, Larsson A, Hansson LO, Flodin M, Nordin G, et al. The revised Lund-Malmö GFR estimating equation outperforms MDRD and CKD-EPI across GFR, age and BMI intervals in a large Swedish population. Clin Chem Lab Med. 2014;52(6):815-24. doi: 10.1515/cclm-2013-0741.

3. Hatløy A, Torheim LE, Oshaug A. Food variety--a good indicator of nutritional adequacy of the diet? A case study from an urban area in Mali, West Africa. Eur J Clin Nutr. 1998;52(12):891-8. doi: 10.1038/sj.ejcn.1600662.

**Supplementary Figure and Tables**

Supplementary Figure 1: Flow chart of patient inclusion in the study. Abbreviation: eGFR, estimated glomerular filtration

Supplementary Table 1: Dosage of sodium zirconium cyclosilicate (SZC) in the first 48 to 72 hours (Week 0)

Supplementary Table 2: Dosage of sodium zirconium cyclosilicate (SZC) during after the first 48 to 72 hours in the study until the end of Week 2

Supplementary Table 3: Servings included in food basket according to food category

Supplementary Table 4: Dosage of sodium zirconium cyclosilicate (SZC) during week 3 to 6

Supplementary Table 5: Maximum and minimum values of plasma potassium during the study (n=26)

Supplementary Table 6: Quality of life during the study (n=25)

Supplementary Table 7: Results related to renal treatment satisfaction questionnaire (n=26)

**Supplementary Figure 1**

**Supplementary Table 1:** Dosage of sodium zirconium cyclosilicate (SZC) in the first 48 to 72 hours (Week 0)

| **In the first 48 hours** | **After 48 hours** | | |
| --- | --- | --- | --- |
| SZC dose | PK (mEq/L) | After 48 hours - Current SZC Dose | |
|  |  | 10 g 3x/day | Special condition |
| 10 g (3 times /day) | 3.0 to 3.4 | 5 g every other day |  |
|  | 3.5 to 4.5 | 5 g/day |  |
|  | 4.5 to 5.0 | 10 g/day |  |
|  | 5.1 to 6.5 | Continue 10 g 3x/day | New sample 24 h later |
|  | ≥ 6.5 | Discontinue^1^ |  |
| **48 to 72 hours** | **After 48 to 72 hours** (only for patients with PK ≥5.1 after 48h) | | |
| 10 g (3 times /day) | PK (mEq/L) | SZC dose | |
|  | 3.0 to 3.4 | 5 g every other day |  |
|  | 3.5 to 4.5 | 5 g/day |  |
|  | 4.5 to 5.0 | 10 g/day |  |
|  | 5.1 to 6.5 | Discontinue^2^ |  |
|  | ≥ 6.5 | Discontinue^1^ |  |

Abbreviations: SZC, sodium zyrconium cyclosilicate; PK,plasma potassium (fasting conditions).

^1^Drug to be discontinued if plasma K < 3.0 mEq/L or > 6.5 mEq/L at any time and rescue treatment should start.

^2^ If normokalemia is not achieved after 72 hours, rescue treatment should start.

**Supplementary Table 2:** Dosage of sodium zirconium cyclosilicate (SZC) after the first 48 to 72 hours in the study until the end of Week 2

|  | **Healthy low potassium diet (Week 0 to the end of Week 2)** | | |
| --- | --- | --- | --- |
| PK (mEq/L) | Current SZC Dose | | |
|  | 5 g every other day | 5 g/day | 10 g/day |
| 3.0 to 3.4 | Discontinue | 5 g every other day | Reduce to 5g/day |
| 3.5 to 5.0 | No change | No change | No change |
| 5.1 to 6.5 | Increase to 10 g/day | Increase to 10 g/day | Maintain 10 g/day |
| ≥ 6.5 | Discontinue^1^ | Discontinue1 | Discontinue^1^ |

Abbreviations: SZC, sodium zyrconium cyclosilicate; PK, plasma potassium (fasting conditions).

^1^Drug to be discontinued if plasma K < 3.0 mEq/L or > 6.5 mEq/L at any time and rescue therapy should start.

**Supplementary Table 3:** Servings included in food basket according to food category

| **Food category** | **Serving** |
| --- | --- |
| **Fruit** | |
| Apple | 1 medium unit (150 g) |
| Banana | 1 medium unit |
| Mango | ½ unit (100 g) |
| Persimmon | 1 medium unit (110 g) |
| Pineapple | 1 medium slice (85 g) |
| Pear | 1 medium (130 g) |
| Peach | 1 medium (80 g) |
| Plum | 1 medium (50 g) |
| Strawberry | 10 small units (120 g) |
| Berries | 1 cup (100 g) |
| Banana | 1 medium (80 g) |
| Melon | 1 medium slice (100 g) |
| Watermelon | 1 medium slice (200 g) |
| Orange | 1 unit (130 g) |
| Clementine / Tangerine | 1 unit (80 g) |
| Kiwi | 1 unit (70 g) |
| Grapes | 10 units (80 g) |
| Avocado | ¼ unit |
| **Vegetables** | |
| Potato | 2 medium units (300 g) |
| Carrot | 1 medium unit (60 g) |
| Zucchini | 1 unit (200 g) |
| Pumpkin | 1 cup (120 g) |
| Eggplant | 1/2 unit (250 g) |
| Peas | 1 cup (120 g) |
| Beet | 1 unit medium (120 g - cooked) |
| Tomato | 1 medium unit (100 g) |
| Cucumber | 1/2 medium unit (30 g) |
| Cabbage | 1 unit (200 g) |
| Radishes | 1 cup (120 g) |
| Cale | 1/2 unit (250 g) |
| Broccoli | 1 cup (120 g) |
| Cauliflower | 1 cup (120 g) |
| Paprika | 1 unit |
| Brussel sprouts | 5 units |
| **Legumes** | |
| Peas | 1 cup (120 g) |
| Corn | 1 medium |
| Chickpeas | 1 cup (120 g) |
| Lentils | 1 cup (120 g) |
| **Nut** (roasted unsalted) | |
| Brazilian nuts | 3 medium units (20 g) |
| Cashew nuts | 3 medium unit (20 g) |
| Almond | 3 unit (20 g) |
| Hazel nut | 6 units (20 g) |
| **Whole cereal** | |
| Whole bread | 2 slices |
| Oats | 1 cup (60 g) |
| Quinoa | 1 cup |
| Whole wheat pasta (cooked) | 1 cup |
| Whole rice (cooked) | 1 cup |
| **White meat, fish** (raw) or **egg** | |
| Chicken breast filet | 1 small file (50 g) |
| Chicken thigh | 1 small unit (50 g) |
| Salmon | 1/2 file (50 g) |
| Cod | 1/2 file (50 g) |
| Egg | 2 units |

**Supplementary Table 4:** Dosage of sodium zirconium cyclosilicate (SZC) during Week 3 to 6

|  | **Healthy PBD diet (Week 3 to 6)** | | |
| --- | --- | --- | --- |
| PK mEq/L | Current SZC Dose | | |
|  | 5 g every other day | 5 g/day | 10 g/day |
| 3.0 to 3.4 | Discontinue | 5 g every other day | Reduce to 5g/day |
| 3.5 to 5.0 | No change | No change | No change |
| 5.1 to 6.5 | Increase to 10 g/day | Increase to 10 g/day | Maintain 10 g/day |
| ≥ 6.5 | Discontinue^1^ | Discontinue^1^ | Discontinue^1^ |

SZC, sodium zyrconium cyclosilicate; PK,plasma potassium (fasting conditions).

^1^Drug to be discontinued if plasma K < 3.0 mEq/L or > 6.5 mEq/L at any time and rescue therapy should start.

**Supplementary Table 5:** Maximum and minimum values of plasma potassium during the study (n=26)

| PK (mEq/L) | Week 0 | Week 0  +48 to 72 hours | Week 2 | Week 3 | Week 3  +48 hours | Week 5 | Week 6 |
| --- | --- | --- | --- | --- | --- | --- | --- |
| Minimum  Maximum | 5.1  6.1 | 3.8  4.9 | 4.0  5.5 | 4.0  5.5 | 4.2  5.9 | 3.9  5.8 | 3.9  5.7 |

Abbreviation: PK,plasma potassium (fasting conditions).

**Supplementary Table 6:** Quality of life during the study (n=25)^1^

|  | **Week 0**  **Baseline^1^** | **Week 3**  **Before PBD^1^** | **Week 6**  **End PBD^2^** | **P^3^** |
| --- | --- | --- | --- | --- |
| Physical Functioning | 79.8±27.5 | 80.6±27.7 | 86.2±18.0^4,5^ | 0.042 |
| Role limitation | 81.0±34.8 | 80.0±35.4 | 80.0±36.8 | 0.97 |
| Bodily pain | 81.7±24.5 | 85.6±22.5 | 84.4±20.8 | 0.26 |
| General health | 61.8±19.2 | 63.0±20.7 | 63.6±21.0 | 0.78 |
| Vitality (energy fatigue) | 67.8±18.5 | 70.6±15.0 | 69.2±17.0 | 0.75 |
| Social functioning | 87.6±19.6 | 92.6±12.8 | 90.0±19.0 | 0.16 |
| Role functioning/emotional | 85.3±27.3 | 84.0± 3.5 | 85.3±30.0 | 0.96 |
| Mental health (emotional well-being) | 79.8±14.4 | 85.2±13.4 | 85.0±12.0 | 0.16 |

^1^One patient did not complete one of the quality-of-life questionnaires, which impeded including the entire sample in the analysis.

^2^Data described as mean ±standard deviation.

^3^Repeated measures ANOVA.

^4^Post-hoc analysis with Bonferroni correction.

^5^Indicate P < 0.05 for the comparison between Week 6 to Week 0

**Supplementary Table 7:** Results related to renal treatment satisfaction questionnaire (n=26)

| Question | **Week 0**  **Baseline^1^** | **Week 3**  **Before PBD^1^** | **Week 6**  **End PBD^1^** | **P**^2^ |
| --- | --- | --- | --- | --- |
| 1. Satisfaction with current treatment | 5.4±0.9 | 5.6±0.6 | 5.7 ±0.6 | 0.20 |
| 2. Satisfaction with control over renal condition | 5.4±0.9 | 5.5±0.6 | 5.6±0.6 | 0.59 |
| 3. Eventual side-effects from the treatment | 4.8±1.7^a^ | 5.1±1.3 | 5.2±1.2 | 0.15 |
| 4. Flexibility of treatment | 5.1±1.3 | 5.2±1.2 | 5.5±0.7 | 0.09 |
| 5. How comfortable do you feel with the treatment | 5.2±1.1 | 5.1±1.0 | 5.5±0.7 | 0.09 |
| 6. How easy has it been to adapt to the treatment | 5.3±1.1 | 5.4±0.8 | 5.7±0.5 | 0.15 |
| 7. Satisfaction with the freedom afforded by the treatment | 5.3±1.1 | 5.4 ±0.8 | 5.6±0.5 | 0.24 |
| 8. Satisfaction on your knowledge on understanding kidney disease | 4.9±1.2 | 4.8±1.2 | 5.1±1.3 | 0.29 |
| 9. Satisfaction your treatment takes | 5.3 ±1.0 | 5.3±0.8 | 5.5±0.7 | 0.14 |
| 10. Discomfort or pain involved with treatment | 5.0±1.6 | 5.3±0.8 | 5.5 ±0.8 | 0.22 |
| 11. How well treatment fits in with lifestyle | 5.2±1.4 | 5.5±0.8 | 5.7 ±0.4 | 0.09 |
| 12. Would you recommend this treatment to others? | 5.3±1.4 | 5.6±0.7 | 5.9±0.3 | 0.054 |
| 13 Satisfaction to continue with present treatment | 5.3±1.2 | 5.7±0.6 | 5.9±0.4^3,4^ | 0.04 |

^1^Data described as mean ±standard deviation

^2^Repeated measures ANOVA test.

^3^Post-hoc analysis with Bonferroni correction

^4^Indicate P < 0.05 for the comparison between Week 6 to Week 0
